# Hyperammonemia and Systemic Inflammatory Response Syndrome Predicts Presence of Hepatic Encephalopathy in Dogs with Congenital Portosystemic Shunts

**DOI:** 10.1371/journal.pone.0082303

**Published:** 2014-01-02

**Authors:** Mickey S. Tivers, Ian Handel, Adam G. Gow, Vicky J. Lipscomb, Rajiv Jalan, Richard J. Mellanby

**Affiliations:** 1 Department of Veterinary Clinical Sciences, Royal Veterinary College, Hatfield, Hertfordshire, United Kingdom; 2 Royal (Dick) School of Veterinary Studies and The Roslin Institute, The University of Edinburgh, Roslin, Midlothian, United Kingdom; 3 UCL Institute for Liver and Digestive Health, UCL Medical School, London, United Kingdom; 4 MRC Centre for Inflammation Research, University of Edinburgh, Edinburgh, United Kingdom; Institute of Hepatology, Foundation for Liver Research, United Kingdom

## Abstract

Hepatic encephalopathy (HE) is an important cause of morbidity and mortality in patients with liver disease. The pathogenesis of he is incompletely understood although ammonia and inflammatory cytokines have been implicated as key mediators. To facilitate further mechanistic understanding of the pathogenesis of HE, a large number of animal models have been developed which often involve the surgical creation of an anastomosis between the hepatic portal vein and the caudal vena cava. One of the most common congenital abnormalities in dogs is a congenital portosystemic shunt (cpss), which closely mimics these surgical experimental models of HE. Dogs with a cPSS often have clinical signs which mimic clinical signs observed in humans with HE. Our hypothesis is that the pathogenesis of HE in dogs with a cPSS is similar to humans with HE. The aim of the study was to measure a range of clinical, haematological and biochemical parameters, which have been linked to the development of HE in humans, in dogs with a cPSS and a known HE grade. One hundred and twenty dogs with a cPSS were included in the study and multiple regression analysis of clinical, haematological and biochemical variables revealed that plasma ammonia concentrations and systemic inflammatory response syndrome scores predicted the presence of HE. Our findings further support the notion that the pathogenesis of canine and human HE share many similarities and indicate that dogs with cPSS may be an informative spontaneous model of human HE. Further investigations on dogs with cPSS may allow studies on HE to be undertaken without creating surgical models of HE thereby allowing the number of large animals used in animal experimentation to be reduced.

## Introduction

Hepatic encephalopathy (HE) is a neuropsychiatric syndrome which occurs in patients with significant liver disorders [1]. The clinical signs of overt HE range from anxiety, lethargy and apathy to gross disorientation and coma [2]. Hepatic encephalopathy has been estimated to occur in approximately 30-45% of patients with cirrhosis and 10-50% of patients with transjugular intrahepatic portosystemic shunt, while minimal HE has been estimated to affect approximately 20-60% of patients with liver disease [3]. The estimated prevalence of chronic liver disease and cirrhosis in the United States is approximately 5.5 million cases and in 2003 there were over 40 000 patients hospitalized for a primary diagnosis of HE, resulting in total charges of approximately $932 million [3]. In addition, trends over the past 10 years suggest that the burden of HE is increasing, as indicated by increases in hospital admissions and higher charges per stay [3].

Due to the significant morbidity and mortality associated with HE, a large number of studies have been undertaken which have examined the pathogenesis of this disorder. Whilst ammonia has long been incriminated in the pathogenesis of HE in humans [4], reports of an absolute correlation between blood ammonia concentrations and severity of HE are not consistent [5]; indeed, it is well recognised that some patients with severe HE have normal ammonia concentrations [6]. Furthermore, two controlled trials of ammonia administration to patients with cirrhosis did not document development of HE [7,8]. 

This has prompted additional research, which has focussed on understanding the role of other contributing factors which may allow hyperammonemia, in the setting of advanced liver disease, to cause HE in a high proportion of patients. Numerous studies have implicated other factors such as manganese [9], hyponatremia [10] and inflammation in the development of HE [5,11]. In terms of inflammation, a strong positive correlation between circulating levels of TNFα and severity of HE has been shown in patients with cirrhosis [12,13]. In addition, induction of hyperammonemia by administration of an amino acid solution to cirrhotic patients without overt HE resulted in the development of HE only in the presence of a systemic inflammatory response [14]. These findings suggest that inflammation, particularly mediated by TNFα, plays a crucial role in the neuropsychological effects of hyperammonaemia in patients with liver disease. Thus, the interaction between ammonia and inflammation/ infection appears to be crucial in the development of HE although the precise mechanism of how the interaction induces HE is unclear [11,15,16].

Due to the large economic and social burden of HE and that the pathophysiology of HE is incompletely understood, a number of animal models of HE have been developed. These typically involve inducing disease in healthy rodents or larger animals such as dogs by invasive surgical or chemical treatments. Portocaval anastomosis is the most widely used rodent model of HE [17]. There are a number of problems with this approach, including variations between studies in surgical techniques and expertise, failure to induce the typical changes in astrocytes observed in humans with HE, and inability to consistently induce overt encephalopathy [17]. Bile duct ligation is another model but animals rarely develop overt HE [17,18]. Hepatic encephalopathy can also be induced in rats by chronic hyperammonemia induced by either ammonium-acetate supplemented diet, parenteral administration of ammonium or urease treatment [17].

Large animal models of HE have played an important role in our understanding of HE as the syndrome was first described in the 1890’s by Pavlov who observed that dogs developed a behavioural syndrome following the formation of a surgical shunt diverting blood away from the portal vein into the inferior vena cava [11]. This approach remains one of the most widely used models of HE and normally involves the creation of an end-to-side portocaval shunt, which may be followed by procedures such as ligation of the hepatic arteries or partial hepatectomy [19,20]. This is typically done in pigs, but the surgical creation of portocaval shunts in normal dogs is also a well-established model of HE [21].

In contrast to experimentally induced HE in dogs, spontaneous HE is well recognised in client-owned dogs with naturally occurring liver diseases [22]. It can develop as a complication of primary liver disease and secondary portal hypertension or as consequence of congenital vascular abnormalities termed portosystemic shunts (cPSS), which link the portal vein directly with the systemic circulation [22]. Congenital portosystemic shunt is one of the most common congenital abnormalities diagnosed in dogs [23]. Dogs with cPSS usually have a single, extra-hepatic vessel which links the portal vein and the caudal vena cava, although a range of vascular abnormalities have been described [24]. In contrast to healthy dogs with surgically created portocaval shunts, dogs with a cPSS can present with signs that are similar to HE in humans ranging from mild lethargy to ataxia, disorientation and coma [22,25]. Dogs can be screened with good sensitivity and specificity for cPSS by measurement of serum bile acids and ammonia [26] and the abnormal vessel can be definitively identified in almost all cases by either abdominal ultrasonography, computed tomography or magnetic resonance imaging [27-29].

We hypothesised that dogs with spontaneous cPSS would be a good model for human HE. This hypothesis is supported by our recent findings that dogs with cPSS have increased whole blood concentrations of manganese and that dogs with a cPSS have higher serum concentrations of C-reactive protein compared to dogs with a cPSS which were asymptomatic, observations which are similar to studies in human HE [9,11,30,31]. We investigated this hypothesis by measuring a range of clinical, haematological and biochemical variables, including plasma ammonia and sodium concentrations and systemic inflammatory response syndrome (SIRS) scores, in 120 dogs with a cPSS both with and without clinical signs of HE. We found that both ammonia and SIRS scores predicted the presence of HE in dogs with a cPSS. Our findings further demonstrate the similarities between human and canine HE and provide support for the concept that additional studies on canine HE may not only provide mechanistic and therapeutic insights into human HE but may also allow the scientific community an opportunity to reduce the number of large animals used in experimentation. 

## Results

### Signalment

One hundred and twenty dogs were eligible for inclusion in the study. Thirty-three were entire females, 18 neutered females, 47 entire males and 22 neutered males. Their ages ranged from 73 days to nearly 12 years with a median age of 333 days [highly skewed to right, mean was 649, 1^st^ quartile 156, 3^rd^ quartile 806]. The breeds of the dog included in the study are shown in table 1. Ninety dogs had no clinical signs of HE and the remaining 30 dogs had clinical signs consistent with HE. 

**Table 1:** Breeds included in the study.

**Breed**	**count**
Basset hound	1
Bichon Frise	5
Border Collie	2
Border Terrier	2
Cairn terrier	1
Cairn Terrier	2
Cavalier King Charles Spaniel	1
Chihuahua	1
Cocker Spaniel	5
Crossbreed	9
Flat Coat Retriever	1
Golden Retriever	3
Great Dane	1
Hovawart	1
Irish Setter	2
Irish Water Spaniel	1
Jack Russel	5
Jack Russell Terrier	1
Labrador	7
Lhasa Apso	2
Maltese Terrier	1
Minature Schnauzer	1
Miniature Dachshund	1
Miniature Poodle	2
Miniature Schnauzer	7
Norfolk Terrier	5
Papillon	1
Pug	3
Rhodesian Ridgeback	1
Scottish Terrier	1
Shetland Sheepdog	1
Shih Tzu	9
Springer Spaniel	1
Staffordshire Bull Terrier	1
Tibetan Terrier	1
Weimaraner	1
West Highland White Terrier	17
Yorkshire Terrier	13

### Uni-variable selection

The mean and standard deviation of the 12 variables examined in this study are listed in table 2. Initially, 17 variables (12 measured plus 5 derived) were screened for their ability to predict the presence of HE in which 11 were identified as candidates with a univariable likelihood ratio test P-value<=0.2. Of these total WBC concentration and the derived indicator variable (WBC2) were highly correlated with the total neutrophil concentration so were excluded. Details of univariable selection are shown in table 3. 

**Table 2:** Mean and standard deviation (brackets) of clinical, haematological and biochemical variables in dogs without and with HE.

**Variable**	**No HE**	**HE**
Ammonia μmol/l	152.6 (101.6)	225.8 (132.0)
PCV %	41.4 (6.7)	39.8 (6.7)
Na mmol/l	148.3 (3.8)	149.7 (3.9)
Lymphocytes x10^9^/l	3.2 (1.6)	2.7 (1.4)
Neutrophils x109/l	9.2 (3.4)	12.5 (6.9)
Monocytes x10^9^/l	0.9 (0.6)	1.6 (1.4)
WBC x10^9^/l	14.1 (4.5)	17.7 (8.2)
RBC x10^12^/l	6.7 (1.1)	6.4 (1.1)
Temperature °C	38.3 (0.5)	38.5 (0.6)
Heart rate /min	113.4 (30.4)	120.2 (35.5)
Respiratory rate /min	29.2 (12.5)	36.1 (18.0)
K mmol/l	4.6 (0.4)	4.5 (0.4)

**Table 3:** Univariable analysis testing individual variable’s ability to predict presence of HE (‡ variables are binary variables based on the SIRS score components – see methodology).

**Variable**	**Likelihood ratio test P-Value**	Candidate for multivariable model development (*)
Ammonia μmol/l	0.0029	*
SIRS	0.0114	*
PCV %	0.2543	
Na mmol/l	0.0964	*
Lymphocytes x10^9^/l	0.1006	*
Neutrophils x109/l	0.0012	*
Monocytes x10^9^/l	0.0008	*
WBC x10^9^/l	0.0044	(Excluded as highly correlated with neutrophil count)
RBC x10^12^/l	0.1139	*
Temperature °C	0.0563	*
Heart rate /min	0.3180	
Respiratory rate /min	0.0271	*
K mmol/l	0.3703	
RR2‡	0.2095	
HR2‡	0.2633	
WBC2‡	0.0651	(Excluded as highly correlated with neutrophil count)
Temp2‡	0.2633	

### Multivariable analysis

Ammonia, lymphocyte, neutrophil and monocyte concentrations were statistically significant predictors of the presence of HE (table 4). When the SIRS score was included as a linear predictor and its components excluded as individual candidate variables SIRS score and ammonia level were significant predictors (table 5). The predictive power of both of these models was at least acceptable with area under ROC curves of 0.83 and 0.71 respectively.

**Table 4:** Multivariable model predicting presence of HE developed from all candidate variables excluding SIRS score.

**Variable**	**Estimate**	**Odds Ratio**	**Std. Error**	**P-Value**
Ammonia	0.0063	1.0063	0.00255	0.0136
Na	0.1105	1.1168	0.07556	0.1436
Lymphocytes	-0.6892	0.5020	0.22193	0.0019
Neutrophils	0.1551	1.1678	0.07027	0.0273
Monocytes	0.7958	2.2161	0.39006	0.0413
RBC	-0.4947	0.6097	0.25707	0.0543

**Table 5:** Multivariable model predicting presence of HE developed from SIRS score and independent candidate variables.

**Variable**	**Estimate**	**Odds Ratio**	**Std. Error**	**P-Value**
Ammonia	0.0050	1.00497	0.0019	0.00979
SIRS score	0.4782	1.61324	0.2314	0.03876

**Figure 1:**
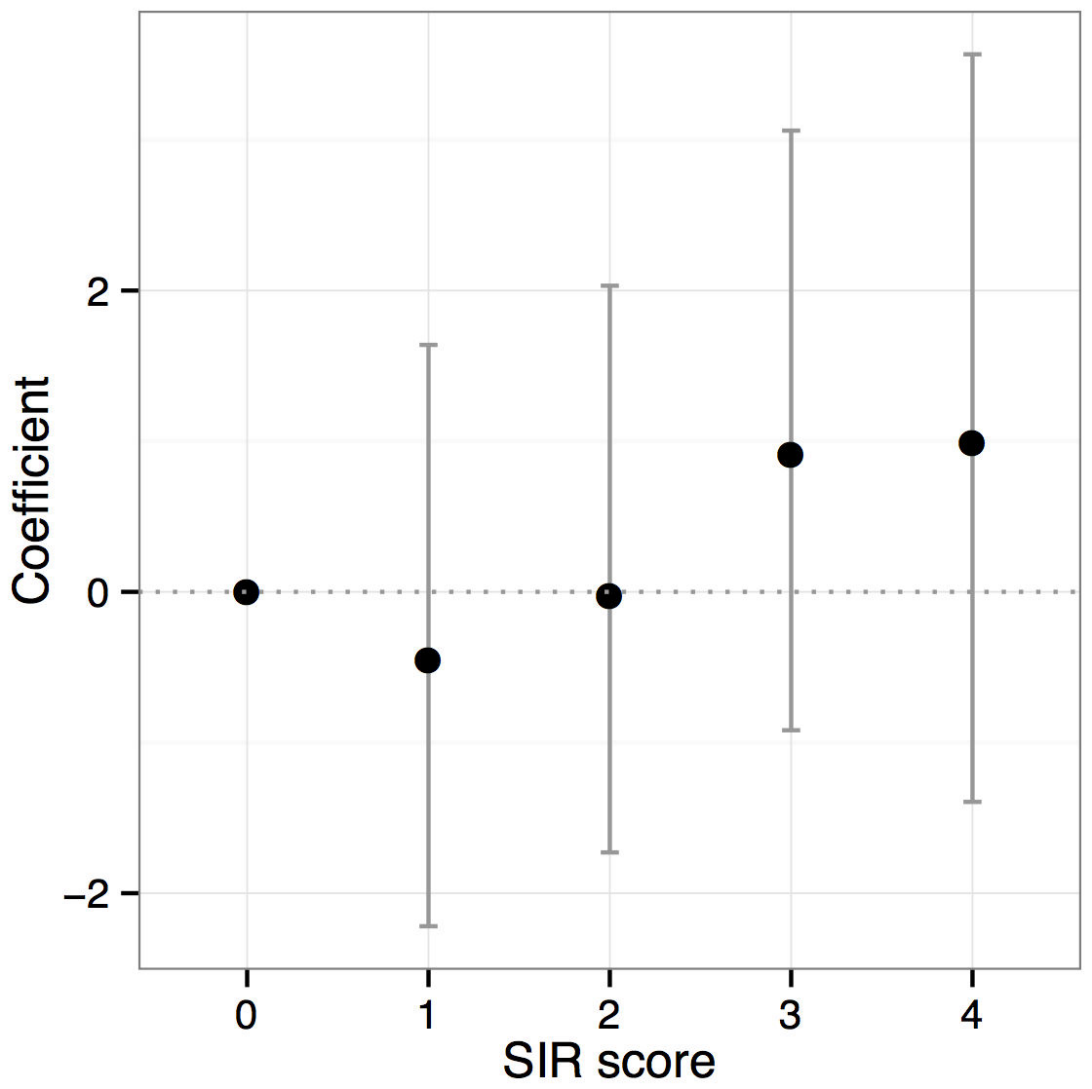
SIRS score plotted against log (odds ratio) of HE relative to reference value of SIRS 0.

The relationship between SIRS score and probability that a dog had HE is shown in Figure 1 where SIRS score is plotted against the logistic regression model coefficient (i.e. the log odds ratio of HE at that score relative to odds at score zero) with 95% confidence interval. The log-odds ratio increases overall with increasing SIRS score although there was an initial decrease between SIRS scores 0 and 1. 

The further model using dichotomised SIRS score combined with remaining candidate variables identified a SIRS score greater than or equal to 3 as best dichotomous predictor of HE (see table 6). Ammonia concentration was also a significant predictor in this model with a coefficient and odds ratio similar to the other models and an area under the ROC curve of 0.73.

**Table 6:** Multivariable model predicting presence of HE developed from dichotomised SIRS score and independent selected variables.

**Variable**	**Estimate**	**Odds Ratio**	**Std. Error**	**P-Value**
Ammonia	0.0043	1.0043	0.0019	0.0260
Na	0.107	1.1131	0.0649	0.0988
RBC	-0.368	6.9201	0.2262	0.1036
SIRS >= 3	1.097	2.9953	0.5182	0.0343

### Follow up on encephalopathic dogs post treatment

To further probe the role of ammonia and SIRS in the development of HE, we investigated how these variables changed in the dogs with hepatic encephalopathy following successful treatment of HE. From the cohort of 30 dogs with HE, 25 were asymptomatic after surgical attenuation of the anomalous vessel at a mean time follow up time of 3.5 months after surgery. In the 25 dogs whose HE resolved following surgery, the plasma ammonia concentration significantly dropped from a pre-treatment median concentration of 251.1µmol/l to a post-treatment median of 79.5µmol/l (p=0.007, n=18). There was no significant difference in SIRS before and after treatment (pre mean 2.12 median 2, post mean 1.57 median 2; p=0.13, n=14). 

## Discussion

The central finding of this study is that both ammonia and inflammation predicts the presence of HE. Whilst ammonia has long been established to be increased in dogs with a cPSS [26], its role in the development of HE has received limited attention, particularly in the context of a cPSS [32]. This study has clearly shown that in dogs, plasma ammonia is predictive for the presence of HE, observations which are similar to studies in humans with HE [6,13]. Ammonia is widely considered to initiate HE through altered astrocytes function as these are the main cells in the brain that can metabolise ammonia. The conversion of glutamate and ammonia to glutamine results in osmotic stress leading to astrocyte swelling, cerebral oedema and intracranial hypertension [33].

However, it is important to acknowledge that several dogs with HE had a normal plasma ammonia concentration. This finding highlights that hyperammonemia is not a universal finding in dogs with a cPSS and HE and suggests that other factors are likely to be important in the initiation of HE in these dogs. Again, this finding is similar to studies in humans which have found that although ammonia is frequently raised in patients with HE, there is an imperfect correlation between ammonia and severity of HE signs [5]. There is a substantial overlap between ammonia concentrations and various grades of HE with some patients with HE having normal ammonia concentrations [6,13]. This result is also consistent with the observation that dogs with urea cycle enzyme deficiency have increased plasma ammonia concentrations but do not typically developing clinical signs of HE [34]. It is also supported by the finding that humans, when administered ammonium chloride, do not develop classical signs of HE [35]. 

The additional main finding of this study is that a high SIRS score was predictive of HE in dogs with a cPSS. This finding is consistent with studies in humans with liver disease and HE [36-39]. The mechanism by which a SIRS may influence the development of HE is unclear although the presence of a SIRS has been shown to exacerbate the neuropsychological effects of induced hyperammonemia in humans [14]. Our study did not find any evidence of an additive effect of ammonia and inflammation, rather we found that HE was associated with the presence of inflammation and hyperammonemia as independent, non-interacting events. 

The finding that ammonia decreases in dogs with HE whose encephalopathic signs resolved following treatment, further supports the notion that ammonia plays a key role in the development of HE. The observation that the mean SIRS score decreased post successful treatment of HE is compatible with the concept that inflammation is important in development of HE. The lack of statistical significance associated with the decrease of SIRS score following treatment may be a consequence of statistical power as the number of paired samples was lower than in the ammonia analysis. Also, the 4 point, categorical nature of the SIRS score means that it lacks sensitivity to detect of changes in inflammation. For example, the temperature, heart rate, respiratory rate and white cell count may have all decreased following treatment but if they did not cross the cut offs used, the SIRS score would not register these changes.

The finding that plasma sodium concentrations did not predict the presence of HE is noteworthy. The presence of hyponatremia is well recognised in humans with liver diseases [10]. In patients with cirrhosis the presence of hyponatremia has been associated with increased risk of developing electroencephalographic abnormalities and HE [40-42]. In addition, the presence of hyponatremia identifies patients with HE that are more resistant to treatment with lactulose [43]. The mechanism by which hyponatriema exacerbates HE is unclear although astrocyte swelling due to reduced osmolality of the extracellular fluid is widely postulated to be important [44,45]. The lack of association between sodium concentrations and HE in dogs with a cPSS may reflect the fact that hyponatremia is only rarely diagnosed in dogs with a cPSS. Hyponatremia may be a rare finding in dogs with a cPSS since portal hypertension and ascites does not occur in dogs unless there is a concurrent primary hepatopathy. 

The cross sectional design of this study does not allow us to establish that ammonia and inflammation initiate HE in dogs with a cPSS. Longitudinal and provocative testing would be required to further investigate whether ammonia and inflammation play a causative role in spontaneous HE in dogs with a cPSS. In addition, we did not evaluate all other factors which have been linked to HE in dogs such as manganese [30], gastrointestinal derived endogenous benzodiazepines [46], hypercortisolism [47] and altered tryptophan metabolism [48]. However, our work clearly demonstrates that both ammonia and inflammation predicts the presence of HE in dogs with a cPSS and provides further evidence that dogs with a cPSS are a good spontaneous model of human HE, notably type B HE [1,49]. Further studies into the pathogenesis of HE in dogs with a cPSS may offer additional insights into the biology of HE in humans and could potentially reduce the numbers of large animals used in animal experimentation. 

## Methods

Consecutive cases of dogs with a cPSS diagnosed at the Royal Veterinary College, London were considered eligible for inclusion in the study. The diagnostic criteria for a cPSS were the identification of a portosystemic shunt on intra-operative mesenteric portovenography and by visual identification of shunting vessel during an exploratory celiotomy. The presence or absence of HE at the time of blood sampling and clinical examination was ascertained by reviewing the case notes. The dog was considered to have HE if it had clinical signs of lethargy, inappropriate behaviour, disorientation, circling, head pressing or seizures [50]. If the dog had no clinical signs of neurological impairment or dysfunction it was considered to not have HE. Details of each dog’s heart rate, respiratory rate, temperature, haematology profile and plasma ammonia, sodium and potassium concentrations were recorded. 

Ammonia was measured using either a spectrophotometer Stasar III Spectrophotometer (Gilford Instrument Laboratories Incorporated, Ohio, USA) or a Jenway 6310 Spectrophotometer (Bibby Scientific Limited, Staffordshire, UK). Haematology was performed using an Abbott Cell-Dyn 3500 (Abbott Diagnostics Limited, Berkshire, UK). Sodium was measured using either a Technicon OPERA Chemistry Analyzer (Bayer Diagnostics, Hampshire, UK) or an IL ILab 600 Chemistry Analyzer (Instrumentation Laboratory, Cheshire, UK). 

Records were available for 120 dogs with complete observation of these 12 variables. A SIRS score was calculated for each dog using the methodology previously described [51,52]. The SIRS score records how many of the following criteria were met for each dog: respiratory rate greater than 20 min-1 [RR2]; heart rate greater than 120 min-1 [HR2]; total WBC less than 6 or greater than 16 x10^9^ L-1 [WBC2] and rectal temperature less than 38.1 or greater than 39.2 degrees Celsius [Temp2]. SIRS scores could hence take a value from 0 to 4. The four, binary, variables recording each of criteria individually (RR2, HR2, WBC2 and Temp2) were also added for each dog.

Prior to development of the multivariable models, the 17 (12 measured plus 5 derived) variables were screened individually to assess their association with the presence of HE. Variables with a likelihood ratio test P-value<=0.2 on logistic regression analysis were selected as candidates for inclusion in the multivariable models. Multi-collinearity (strong correlation of variables that may produce spurious statistical results) was assessed by scatter-plots and Pearson’s product moment correlation.

Two multivariable logistic regression models predicting the presence of HE were then developed; one using all the candidate variables from univariable screening excluding the SIRS score; the other using the ordinal (0-4) SIRS score and those candidate variables that were not components of the SIRS score (RBC, Sodium and Ammonia). Final multivariable models were built using stepwise selection based on Akaike Information Criteria (AIC – a measure of model fit with a penalty for number of parameters used). To reduce the effect of confounding, variables were retained in the multivariable model, even if they were individually statistically non-significant (using a critical p-value of 0.05), if their exclusion altered the coefficients of statistically significant predictors by more than 10%. The validity of including continuously measured variables and the ordinal variable, SIRS score, as linear predictors on the log-odds scale was checked by examination of density/frequency plots of the variables for the HE and non-HE outcomes and by categorisation of the continuous predictors and estimation of coefficients for each category to detect grossly non-linear relationships. Model goodness of fit was assessed using the Hosmer-Lemeshow test. The predictive ability of the models was assessed by estimation of area under the receiver operating characteristic curve. A further model was developed to identify a single optimal cut-off value of SIRS score as a dichotomous predictor of HE using AIC as the model selection criteria. Ammonia concentrations and SIRS were compared before and after treatment by a Wilcoxon matched-pairs test. Data management and statistical analysis used the R statistical system (R Development Core Team (2012)). The study was approved by the Royal Veterinary College Ethics Committee. The owners of the dogs gave permission for their animals to be used in this study. 
